# Clownfishes evolution below and above the species level

**DOI:** 10.1098/rspb.2017.1796

**Published:** 2018-02-21

**Authors:** Jonathan Rolland, Daniele Silvestro, Glenn Litsios, Laurélène Faye, Nicolas Salamin

**Affiliations:** 1 Department of Computational Biology, University of Lausanne, Biophore, Quartier-Sorge, 1015 Lausanne, Switzerland; 2 Swiss Institute of Bioinformatics, Quartier Sorge, 1015 Lausanne, Switzerland; 3 Department of Zoology, University of British Columbia, #4200-6270 University Blvd, Vancouver, BC, Canada; 4 Department of Biological and Environmental Sciences, University of Gothenburg, Carl Skottsbergs gata 22B, Gothenburg 41319, Sweden; 5 Gothenburg Global Biodiversity Centre, Box 461, SE-405 30 Gothenburg, Sweden; 6 Department of Biological Sciences, Simon Fraser University, 8888 University Drive, Burnaby, BC, Canada V5A 1S6

**Keywords:** macroevolution, microevolution, intraspecific, interspecific, positive selection, *RH1*

## Abstract

The difference between rapid morphological evolutionary changes observed in populations and the long periods of stasis detected in the fossil record has raised a decade-long debate about the exact role played by intraspecific mechanisms at the interspecific level. Although they represent different scales of the same evolutionary process, micro- and macroevolution are rarely studied together and few empirical studies have compared the rates of evolution and the selective pressures between both scales. Here, we analyse morphological, genetic and ecological traits in clownfishes at different evolutionary scales and demonstrate that the tempo of molecular and morphological evolution at the species level can be, to some extent, predicted from parameters estimated below the species level, such as the effective population size or the rate of evolution within populations. We also show that similar codons in the gene of the rhodopsin *RH1*, a light-sensitive receptor protein, are under positive selection at the intra and interspecific scales, suggesting that similar selective pressures are acting at both levels.

## 1. Introduction

Understanding the evolutionary process necessitates the integration of multiple biological scales that are rarely studied together. Population biologists have focused their efforts on the study of the variations in allelic frequencies and the mechanisms of evolution of quantitative traits at the population level, what we call microevolution [1–3]. By contrast, palaeontologists and phylogeneticists have been interested in the dynamics of diversification and the rate of phenotypic evolution above or at the level of the species, what is commonly referred to as macroevolution [4,5]. These different timescales challenge our understanding of evolutionary biology and question whether the mechanisms of microevolution could explain the rate of evolution at the macroevolutionary scale. A famous illustration of the difference in rates of evolution observed between timescales is the paradox of stasis, i.e. the discrepancy between the slow morphological evolution in the fossil record and the fast evolution of organisms measured near present [6,7].

Linking micro- and macroevolution remains one of the greatest current challenges in evolutionary biology [8–14] and relatively few empirical studies have provided mechanisms that can explain the two evolutionary scales; such as natural and sexual selection in stick insects [15], selection related to the beak morphology in Darwin finches [16], or sexual preferences related to the colour of cichlids fishes [17]. When comparing micro- and macroevolution dynamics, the rates of evolution are usually predicted to be slower at the macroevolutionary scale than at the microevolutionary scale [12,13]. A first explanation to this observation is that most of the genetic and morphological variation at the intraspecific level can be lost through evolutionary time due to the extinction of locally adapted populations [2]. For instance, at the molecular level, we expect that genetic polymorphism (neutral or under selection) observed at the present time can be lost before being fixed in the species; hence the rate of molecular evolution should be higher within species than among species [18]. At the morphological level, it is also possible that morphological evolution related to environmental fluctuations on short timescales may never accumulate on large timescales because of stabilizing selection [8,13]. At the same time, it has also been proposed that rates of evolution at macroevolutionary scale could potentially be inferred from rates of evolution at microevolutionary scale, as macroevolution corresponds to several rounds of microevolution [6,12,19–22]. Some authors have even proposed that rates of evolution at the species level might be inferred from parameters estimated at the intraspecific level, such as effective population size [12]. Finally, it has been proposed that if there was a continuum of divergence from populations to the species level [23], similar selective pressures (ecological or evolutionary factors) might act at both micro- and macroevolutionary scales. All these predictions still remain to be tested empirically with datasets encompassing morphological and genetic data for a reasonable number of species and individuals within species [13].

Here, we compared the rate of evolution of clownfishes at both intra and interspecific scales. We first constructed two related datasets combining morphological, molecular and ecological features of clownfishes (Pomacentridae, genera *Amphiprion* and *Premnas*) at the species-level and within the species *Amphiprion clarkii*. Then, using newly generated sequences of one gene potentially under selection (*RH1*, implicated in dim light vision in deep sea [24,25]) and five morphological traits important for fish ecology (electronic supplementary material, S1), we tested the three following predictions: (i) rate of evolution should be accelerated within species compared to between species. (ii) Genetic and morphological evolutionary rates at the species level may be inferred with the parameters estimated below the species level, such as the effective population size or the microevolutionary rate. (iii) The major selective forces (i.e. related to water depth here) should be acting similarly at both the population and the species level.

## 2. Results

### (a) Comparison between micro- and macroevolutionary rates

**Figure 1.**
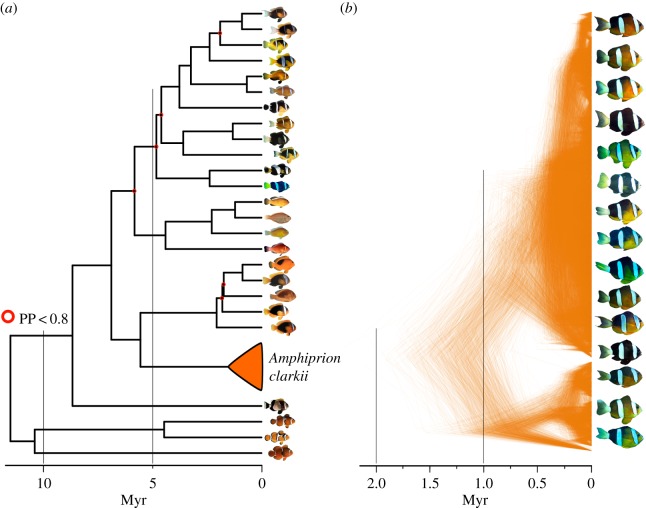
(*a*) Phylogenetic tree showing the relationships between species of clownfishes (macroevolution dataset) and (*b*) coalescence process showing the relationship between 53 individuals of *A. clarkii* (microevolution dataset). Nodes highlighted in red are nodes less supported, with a posterior probability inferior to 0.8. (Online version in colour.)

**Figure 2.**
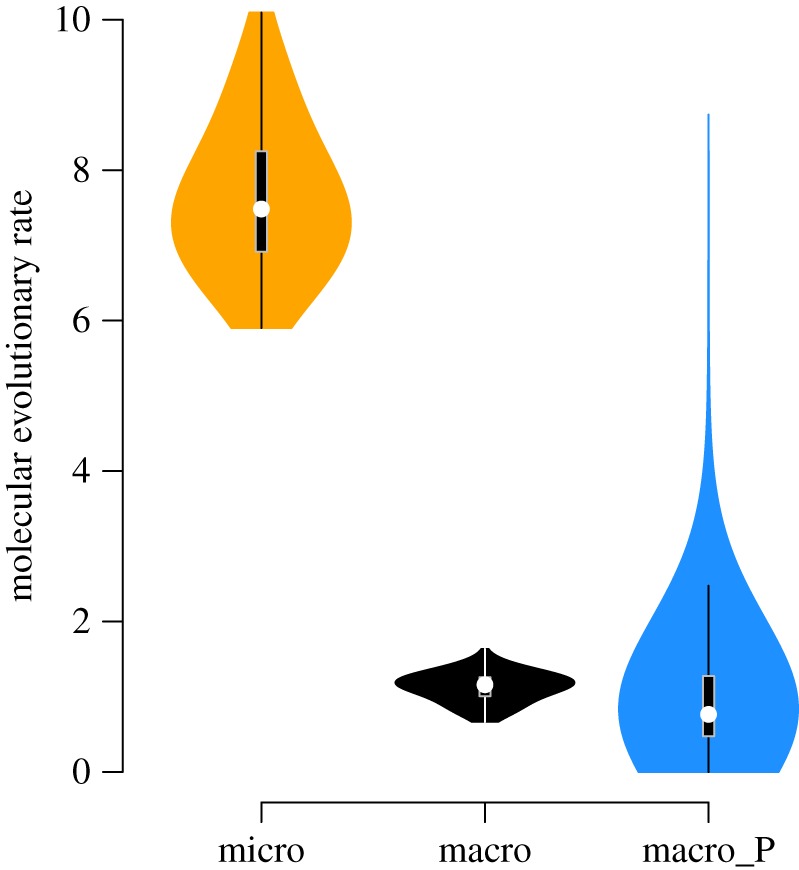
Comparison between the micro- and macroevolutionary rates and the predicted macroevolutionary rate for molecular data (*RH1*). The columns (micro) in yellow and (macro) in black correspond to the estimates of micro- and macroevolutionary rates obtained from RH1 sequences. The column macro_P, in blue, represents the predicted macroevolutionary rate based on *A. clarkii* molecular evolutionary rate described in the Material and methods. This rate was obtained by a subsampling procedure by pruning all but one *A. clarkii* individual in the tree containing all clownfish species and *A. clarkii* individuals. Estimates of molecular evolutionary rate are given in number of mutations × 10^−3^/Myrs for the microevolutionary scale and in number of substitutions × 10^−3^/Myr for macroevolutionary scale. (Online version in colour.)

Using our two datasets of clownfish species and *A. clarkii* individuals (figure 1), we showed that, in line with theoretical expectations [6,18], rates of molecular evolution at the micro- and macroevolutionary scales (*r*_micro_ and *r*_macro_) were substantially different, with higher evolutionary rates within species than among species. This difference was strong for the gene *RH1*, for which we estimated higher rates of molecular evolution within *A. clarkii* (median *r*_micro_ = 7.48 × 10^−3^ [95% confidence interval: 5.90 × 10^−3^, 1.01 × 10^−2^], expressed in number of mutations per million years) than across species of clownfishes (*r*_macro_ = 1.16 × 10^−3^ [6.67 × 10^−4^, 1.64 × 10^−3^] expressed in number of substitutions per million years; figure 2).

### (b) Predicting macroevolution from microevolution

**Figure 3.**
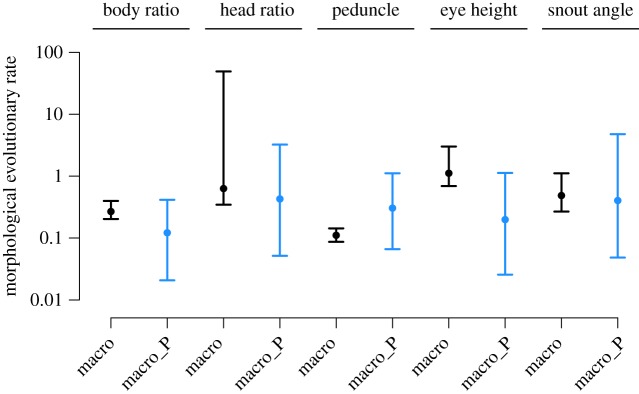
Comparison between the macroevolution rates estimated from empirical data and simulations (based on intraspecific parameters) of the five morphological traits. The columns (macro) in black correspond to the estimates of macroevolutionary rates observed in the data, and (macro_P) in blue, represent the predicted macroevolutionary rate of the simulations based on effective population size, trait variance and generation time (described in details in electronic supplementary material, S2). The dots represent the median and the segment represents the 95% confidence interval of the distribution of rates across the 1000 trees. For each trait, simulated and empirical rates of evolution (*σ*^2^) were obtained from best fitting model chosen only from the empirical data (BM or OU, electronic supplementary material, table S4). The predicted and observed distributions of macroevolutionary rates did not differ significantly in any of the traits (*p* > 0.05). (Online version in colour.)

Our analyses support the hypothesis that macroevolutionary rates can be predicted from microevolutionary rates for both molecular and morphological data (figures 2 and 3). Most of the intraspecific variation linked with neutral or locally adaptive alleles, is likely lost at the macroevolutionary timescale because such alleles are rarely fixed in a large number of populations of a species [2,19]. The reproductive isolation leading to speciation, and the founder effect likely associated with speciation, favours the fixation of these alleles and enables them to keep their imprint at macroevolutionary scales. To test this hypothesis with molecular sequences, we artificially simulated the fixation of one of the haplotypes present in the *A. clarkii* species by pruning all but one *A. clarkii* individual from the intraspecific tree and we estimated the rate of evolution along the single branch leading to *A. clarkii.* This subsampling procedure mimics the loss of intraspecific (neutral or non-neutral) variation of haplotypes through long evolutionary timescale [2,19]. Using this approach, we obtained similar estimates between the predicted rates of molecular evolution of *RH1* estimated with one individual of *A. clarkii* (*r*_macro_P_ = 7.66 × 10^−4^ [4.90 × 10^−9^, 8.74 × 10^−3^]) and the molecular rate estimated at the interspecific level in all clownfish species (*r*_macro_ = 1.16 × 10^−3^ [6.67 × 10^−4^, 1.64 × 10^−3^]; figure 2).

For the five morphological traits, we predicted the rate of evolution at the species level using simulations based on parameters estimated at the intraspecific level, i.e. effective population size and the intraspecific variance of each trait (electronic supplementary material S2 and table S4, figure 3). For all traits, we found no significant difference between the empirical and the predicted rates of evolution across a posterior distribution of 1000 trees (*p* > 0.05, electronic supplementary material, table S4) and regardless of the model selected (Ornstein–Uhlenbeck: OU or Brownian motion: BM). These results indicate that our estimations are robust to uncertainties in model testing, tree topology and branching times (electronic supplementary material, table S4). The simulations also retrieved the best fitting model previously selected on empirical data for four of the five traits (electronic supplementary material, table S4). We show with additional analyses that the small size of the species level phylogeny is not spuriously influencing our model selection (between BM and OU, *pmc* analysis, electronic supplementary material, figure S1), or affecting the size of the confidence intervals around the rate of evolution estimates (*fitcontinuous* analysis [26], electronic supplementary material, table S5). Additional specificity tests also showed no support for a link between micro- and macroevolutionary rates when the effective population sizes are larger or smaller than those estimated from the empirical data (rates are significantly different from the empirical ones, electronic supplementary material, S2 and table S2). This result indicates that the match found between micro- and macroevolutionary rate is not due to a lack of statistical power.

### (c) Comparison between selective pressures between micro- and macroevolutionary scales

At the molecular level, the McDonald–Kreitman test confirmed that the *RH1* sequence was under positive selection (*p* < 0.05, electronic supplementary material, table S6). At the microevolutionary scale, two amino acid sites (L154I and A299S) of the *RH1* gene were found to be under strong positive selection across the posterior distribution of 1000 trees (d*N*/d*S* > 1 and Bayes empirical Bayes probability (BEB) > 0.9, table 1). At the macroevolutionary scale, the same two sites and two additional sites (S164S and L88F) showed a dN/dS ratio greater than one in the *RH1* gene, with high BEB probability (greater than 0.9 for 1000 trees). Two sites (L88F and L154I) were associated with water depth at macroevolutionary scale (Wilcoxon signed-rank tests, *p* < 0.01). At the microevolutionary scale, the relationship between water depth and the two sites under selection (L154I and A299S) was not significant.

**Table 1.** Detection of positive selection in the rhodospin gene (*RH1*) at both micro- and macroevolution timescales. Mean and standard deviation values of the analyses replicated over 1000 trees drawn from a posterior distribution of phylogenetic tree of *A. clarkii* individuals (microevolution), and of all species of clownfishes (macroevolution). The two codons under positive selection in both micro- and macroevolution are italicized. All of these amino acid position have an associated BEB probability greater than 0.9 for all phylogenies.

	amino acid position	BEB probability	*ω* = d*N*/d*S*
macroevolution	*L154I*	*0.99* *±* *2.2* *×* *10^−4^*	*5.61* *±* *0.07*
	*A299S*	*1.00* *±* *7.1* *×* *10^−5^*	*5.63* *±* *0.07*
	S165S	0.94 ± 0.001	5.35 ± 0.07
	L88F	0.93 ± 0.001	5.30 ± 0.07
microevolution	*L154I*	*1* *±* *0*	*8.20* *±* *0.36*
	*A299S*	*1* *±* *0*	*8.20* *±* *0.36*

## 3. Discussion

For decades, researchers have faced the challenge to interpret macroevolutionary dynamics in the light of microevolutionary mechanisms. Only few studies have compared the dynamic of evolution below and above the species level [13,14]. Here, we propose an empirical attempt to compare and link evolutionary rates at both micro- and macroevolutionary scales, and we show that macroevolution can be, at least to some extent, predicted by microevolution.

First, the molecular rates estimated at the microevolutionary scale were strikingly larger than those at the macroevolutionary scale, which is congruent with the trends observed in other studies [13,14] and corroborates well-known evolutionary patterns already described by the paradox of stasis [6].

Second, our results from both molecular and morphological data, suggest that parameters estimated at the intraspecific level, such as the intraspecific variance and the effective population size, can predict the rate of evolution at interspecific level. For molecular data, we used randomizations to simulate the loss of intraspecific genetic variance during the process of evolution, as most of the local adaptations of diverging populations will be deleted through introgression events or bottlenecks associated with the speciation process [2,19]. By pruning lineages and simulating the fixation of a single intraspecific variant at the microevolutionary scale, we were able to predict the rate of molecular evolution estimated for all clownfish species from the rate evolution of *A. clarkii* individuals. These results are consistent with the hypothesis of ‘ephemeral divergence’, previously proposed by Futuyma [2], which stipulates that most of the intraspecific molecular (and trait) variation can be considered as ‘ephemeral’ and should not be observable at higher taxonomic levels. Another link between micro- and macroevolutionary rates was proposed more recently by Hansen & Martins [12], who hypothesized that the tempo of molecular and morphological evolution of species should be directly related to parameters estimated at the intraspecific level, such as the effective population size [22]. Using simulations, we predicted rates of macroevolution based on the trait variance, the generation time and the effective population size estimated at the intraspecific scale, which was consistent with Hansen & Martins' hypothesis*.* Our results were robust to different models of evolution and phylogenetic uncertainties (topology and dating). Overall our analyses demonstrate that the rates of evolution of both molecular and morphological traits between species of clownfishes can be explained by parameters estimated at the intraspecific level, which suggest that likely similar mechanisms are at play across these two scales.

Third, we detected positive selection in the rhodopsin *RH1* gene (light-sensitive receptor protein) for the same two amino acid positions (L154I and A299S) at both micro- and macroevolutionary scales. *RH1* is ubiquitous and well-studied among vertebrates [24], such as cichlids and sharks [24,27,28]. Several key amino acid positions are known to change the spectral specificity of the protein and thus allow organisms to adapt to different light intensity regimes [28]. We identified four amino acid positions of *RH1* gene under positive selection among the clownfish species and two of those four were also present in the *A. clarkii* populations. One of the sites positively selected at both scales (L154I) was also associated with water depth at macroevolutionary scale. We hypothesize that this association with water depth is also present at microevolutionary scale (even if we do not detect it) because in *A. clarkii* L154I covaries with another position (A299S) well known for being linked with light absorption [29–31]. We conclude that both L154I and A299S are two excellent candidates for adaptation to water depth. Our results suggest that molecular changes related to adaptation at the individual level likely cascades to the evolutionary process recorded at the macroevolutionary scale, but further investigation will require much larger sample sizes both in terms of individuals and number of polymorphic sites (e.g. such as in genome wide association studies).

Our approach, applied here on clownfishes, is exploratory and future studies will benefit from using larger molecular data sets for both evolutionary scales and expanding the scope to other traits likely related to adaptation to water depth, such as fin morphology. Our study also simplifies the process of evolution and predicts rates of macroevolution assuming that each incipient species is formed by one haplotype during speciation event. Further studies should investigate if speciation events involving a large amount of genetic diversity will affect the macroevolutionary rates on the long term. As more empirical transdisciplinary studies are expected in the coming years, the next decade will likely see a substantial improvement in the understanding of the links between the different scales of the evolutionary process. Recent literature proposes several directions for future research. First, one of the simplest ways to deal with the problems related to the micro-macroevolution boundary is to take into account intraspecific variance into the reconstruction of phenotypic evolution above the species level [32]. Second, as our results suggested that the evolutionary process from the populations to the species is a continuum (that is tractable numerically), we believe that the remaining gap in our understanding of the overall evolutionary process may not be strictly related to the dichotomy between micro- and macroevolution but rather to other scales, such as the link between individuals and populations levels inside species. To study evolution at multiple scales simultaneously, important developments have been made recently at the crossroad between macroevolution and ecology, such as the individual-based macroevolutionary models related to the unified neutral theory of biodiversity [33,34]. Third, a better understanding of the mechanisms of evolution might also be gained from the field of quantitative genetics focusing on the changes in allelic frequency or phenotype at each generation [35], or on the branching process of populations in function of ecological conditions and population size [36]. Finally, future challenges also concern the understanding of how the phenotypic variance evolves through time [23] in the adaptive landscape [37], and its potential impact on diversification.

## 4. Conclusion

Despite considerable discussion about the potential bridges between the micro- and the macroevolution in the last decades [9,12,38], relatively few studies have proposed an empirical test of theoretical predictions [13,14]. We suggest here that macroevolutionary rates might be inferred using microevolutionary rates in clownfishes. Furthermore, we also propose that the selective processes may act on the same genes (and even codons) at both micro- and macroevolutionary scales. While further studies are necessary to establish the generality of our findings across different organisms, our study on clownfishes provide insights into understanding of the genetic and phenotypic differentiation within and across species.

## 5. Material and methods

### (a) Field sampling, morphological and environmental data

For the microevolutionary scale, we chose *A. clarkii* because we had a good knowledge of its distribution from previous fieldwork [31]. For this species, we were able to collect molecular and morphological information for a total of 53 individuals in the centre of the range, in the Indonesian coral reefs (on the coasts of Bali and Manado, Sulawesi; table 2, electronic supplementary material, figure S5). The Indo-Australian Archipelago region has been previously shown as the main centre of genetic and morphological diversity in clownfishes [31], consistently with the peak of species diversity found in this region for other fishes [39].

**Table 2.** List of the GenBank accession number for the genetic data newly sequenced (for *CR, cytB, RH1*), water depth, and locations of the 53 *A. clarkii* individuals sampled in the field.

isolate	CR	CytB	RH1	depth (m)	location	site	latitude	longitude
GB057	KP764430	KP749692	KP764521	10.4	Tulamben	Noisy Reef	−8.29389	115.61083
GB061	KP764432	KP749694	KP764523	12.5	Tulamben	Noisy Reef	−8.29389	115.61083
GB062	KP764433	KP749695	KP764524	5.5	Tulamben	Noisy Reef	−8.29389	115.61083
GB063	KP764434	KP749696	KP764525	10	Tulamben	Seraya	−8.295	115.61167
GB064	KP764435	KP749697	KP764526	6	Tulamben	Seraya	−8.295	115.61167
GB071	KP764436	KP749699	KP764528	9.5	Tulamben	Melaste	−8.29471	115.60667
GB072	KP764437		KP764529	7.5	Tulamben	Melaste	−8.29471	115.60667
GB075	KP764440	KP749702	KP764532	1.9	Tulamben	Melaste	−8.29471	115.60667
GB079	KP764441	KP749703	KP764533	17.9	Tulamben	Batu Belah	−8.33222	115.64611
GB080	KP764442	KP749704	KP764534	17.9	Tulamben	Batu Belah	−8.33222	115.64611
GB087	KP764445		KP764538	10.2	Tulamben	Batu Belah	−8.33222	115.64611
GB088	KP764446	KP749707	KP764539	13	Tulamben	Batu Belah	−8.33222	115.64611
GB092	KP764450	KP749711	KP764543	7.5	Tulamben	Batu Belah	−8.33222	115.64611
GB009	KP764417	KP749681	KP764506	3.2	Tulamben	Batu Belah	−8.33222	115.64611
GB021	KP764419	KP749683	KP764508	21.6	Tulamben	Batu Belah	−8.33222	115.64611
GB054	KP764428		KP764519	5.2	Amed	Pyramids	−8.33694	115.66056
GB011	KP764418	KP749682	KP764507	3	Nusa Lembongan	Sental	−8.67556	115.52444
GB025	KP764422	KP749686	KP764511	22	Nusa Lembongan	Sental	−8.67556	115.52444
GB159	KP764461	KP749729	KP764557	17.8	Nusa Lembongan	Sental	−8.67556	115.52444
GB162	KP764462	KP749731	KP764558	4.5	Nusa Lembongan	Sental	−8.67556	115.52444
GB163	KP764463	KP749732	KP764559	6.5	Nusa Lembongan	Sental	−8.67556	115.52444
GB023	KP764420	KP749684	KP764509	16.5	Nusa Lembongan	PED (temple)	−8.67167	115.50361
GB143		KP749725	KP764556	14	Nusa Lembongan	Scholar Dasar	−8.67111	115.49806
GB121	KP764456	KP749721	KP764551	14	Pemuteran	Close encounter	−8.12806	114.66694
GB122	KP764457	KP749722	KP764552	8	Pemuteran	Close encounter	−8.12806	114.66694
GB123	KP764458	KP749723	KP764553	14	Pemuteran	Coral garden	−8.13944	114.65472
GB127	KP764459		KP764554	9.8	Pemuteran	Coral garden	−8.13944	114.65472
GB169	KP764464	KP749733	KP764560	11.5	Manado	Tanjung husi II	1.74	125.14389
GB170	KP764465	KP749734	KP764561	12	Manado	Tanjung husi II	1.74	125.14389
GB171	KP764466	KP749735	KP764562	5	Manado	Tanjung husi II	1.74	125.14389
GB172	KP764467		KP764563	18	Manado	Tanjung husi II	1.74	125.14389
GB176		KP749736	KP764564	9	Manado	Tanjung husi II	1.74	125.14389
GB177	KP764468	KP749737	KP764565	8.2	Manado	Tanjung husi II	1.74	125.14389
GB189	KP764471	KP749739	KP764566	8	Manado	Tanjung husi II	1.74	125.14389
GB030	KP764423	KP749687	KP764512	18	Manado	Tanjung husi II	1.74	125.14389
GB031	KP764424		KP764513	15	Manado	Tanjung husi II	1.74	125.14389
GB204	KP764473	KP749742	KP764567	11.5	Manado	Sahaung I	1.74889	125.15972
GB205	KP764474	KP749743	KP764568	11	Manado	Sahaung I	1.74889	125.15972
GB207	KP764475	KP749744	KP764569	7	Manado	Sahaung I	1.74889	125.15972
GB210	KP764476	KP749745	KP764570	5	Manado	Bulu bulu kuning	1.74556	125.14028
GB211	KP764477	KP749746	KP764571	5	Manado	Bulu bulu kuning	1.74556	125.14028
GB035	KP764425		KP764514	4	Manado	Bulu bulu kuning	1.74556	125.14028
GB228	KP764484	KP749752	KP764580	9.5	Manado	House reef	1.74917	125.13667
GB229	KP764485	KP749753	KP764581	9.5	Manado	House reef	1.74917	125.13667
GB230	KP764486	KP749754	KP764582	4.5	Manado	House reef	1.74917	125.13667
GB237	KP764487		KP764583	2	Manado	House reef	1.74917	125.13667
GB036	KP764426	KP749688	KP764515	4.3	Manado	House reef	1.74917	125.13667
GB221	KP764480		KP764576	10	Manado	Batu tiga	1.76972	125.17694
GB223	KP764482	KP749750	KP764578	11	Manado	Batu tiga	1.76972	125.17694
GB224	KP764483	KP749751	KP764579	7.5	Manado	Batu tiga	1.76972	125.17694
GB037		KP749689	KP764516	5.7	Manado	Busa bora	1.7625	125.12861
GB213	KP764478	KP749747	KP764572	6	Manado	Areng kambing	1.76928	125.1794
GB218	KP764479	KP749748	KP764574	10	Manado	Areng kambing	1.76928	125.1794

Once the fishes were spotted during scuba dives, we caught the specimens using clove oil and nets. We recorded the water depth and clipped the anal or caudal fin of the fish for genetic analyses. We also took a picture of the left side of the body before releasing it back into the host anemone (electronic supplementary material, S3, and figure S2). We used these pictures to measure five main axes of body shape variation associated with key aspects of fish ecology: body and head ratios, peduncle factor, eye height and snout angle. These traits were selected because they correspond to standard morphological measurements related to functional aspects of the reef fish's ecology, such as locomotion, sensory abilities or feeding behaviour [40–43] (electronic supplementary material, S1).

For the macroevolutionary scale, we obtained samples from previous fieldwork (Bali, Indonesia; and Madagascar), and from loans from aquariums or research institutions [31] for one individual per species for 26 species of clownfishes including *A. clarkii* (87% of the 30 species of clownfishes). Mean depth for each clownfish species was calculated using the minimum and maximum depth collected in fishbase (http://www.fishbase.org/ [44]; electronic supplementary material, table S3), and our field data for *A. clarkii* (table 2). For morphological information, pictures of the left side of specimens were obtained from museum specimens. In order to obtain intraspecific variance on morphological traits per species, we collected pictures in museum and specialist collections for several individuals per species (total of 307 individuals, with an average of nine individuals per species; min = 2 and max = 31, electronic supplementary material, table S1). Our analysis should not be biased by plasticity between species given that we estimate mean phenotypes using several individuals per species and that clownfish species are morphologically distinct.

### (b) Molecular data

We extracted the DNA of *A. clarkii* individuals using the DNeasy Blood and Tissue kit (Qiagen GmbH, Hilden, Germany), and we also used already extracted DNA of all other clownfish species obtained from a previous study [31]. Using standard protocols, we amplified fragments of the cytochrome B (*cytB*) and mtDNA control region (*CR*) for *A. clarkii* individuals and the rhodopsin gene (*RH1*) for *A. clarkii* individuals and all the other clownfish species (electronic supplementary material, S4). All newly generated sequences have been deposited in the GenBank database (table 2 and electronic supplementary material, table S3).

### (c) Phylogenetic reconstruction

For the macroevolutionary scale, we used 1000 phylogenetic trees of all clownfish species directly from the posterior distribution of trees built with seven nuclear markers of a recently published study [31]. For the microevolutionary scale, we reconstructed the relationships between *A. clarkii* individuals using BEAST 2.1.3 [45] by concatenating the alignments from the *cytB* and *CR* markers. Relationships between individuals within *A. clarkii* were reconstructed using a coalescent prior with constant population size and a strict molecular clock with a uniform prior (electronic supplementary material, S5 and S6). The strict molecular clock was selected with the clock model selection implemented in MrBayes 3.2 [46]. We also chose the best model of substitution using *phyml.test* function in *ape* R package. We obtained a distribution of 1000 ultrametric trees of *A. clarkii* individuals with a root node at a relative date of 1. This distribution revealed that only the three deeper nodes were highly supported in the tree of *A. clarkii* individuals (Posterior probabilities = 1). Recent nodes were less supported, which is expected given that they represent relationships between closely related individuals. To time-calibrate each *A. clarkii* tree, we used several individuals of *A. clarkii* also present in the dated phylogenetic tree at the species level [31]. We used the crown node of all *A. clarkii* individuals that was in common between the two trees to rescale all the branches of the *A. clarkii* tree. We combined each of the 1000 *A. clarkii* trees with each of the 1000 species level trees. Each combination of tree was chosen randomly ensuring that the resulting combined trees were not biased toward certain topologies. We thus obtained a time-calibrated distribution of 1000 topologies encompassing both *A. clarkii* individuals and all the other clownfish species. In the following analyses, micro- and macroevolutionary datasets were considered separately except in the analysis of the molecular rate of evolution where there were combined.

### (d) Estimation of rates of evolution at micro- and macroevolutionary scales

We first estimated whether the rates of molecular evolution were different within and between species. We compared the rate of evolution of the *RH1* gene between *A. clarkii* and all the other clownfish species as a proxy for molecular evolutionary rate (electronic supplementary material, S7). Because *RH1* was potentially under selection, we estimated the rate of substitution by estimating the branch length of a *RH1* tree, while constraining the topology with the dated phylogeny built using neutral genes (*cytB* and *CR*) using the *optim.pml* function of the *phangorn* R package [47]. We then estimated the mean rate of molecular evolution by calculating the ratio between the sum of the branch lengths of *RH1* tree and the sum of the branch lengths of the tree built with neutral genes. This analysis was replicated separately over the two distributions of 1000 trees at micro- and macroevolutionary levels. At microevolutionary scale, the estimates of molecular rates of evolution were obtained directly from the 1000 trees of *A. clarkii* individuals. At macroevolutionary scale, the molecular rate of evolution was obtained from the 1000 species-level trees, but given that each of the 53 *A. clarkii* sequence may represent the *A. clarkii* species in the tree, we ran the analysis for the 53 possible species level trees resulting in 53 000 analyses (= 53 × 1000). We then estimated if rates of evolution were significantly different between the micro- and the macroevolutionary scales by comparing the 95% confidence intervals of the rates, i.e. when the confidence intervals were not overlapping evolutionary rates were significantly different.

We compared micro- and macroevolutionary rates only for molecular data, while morphological rates of evolution were not estimated at the intraspecific level given that individual variation in morphological traits may be due to other factors than evolution (e.g. plasticity, but see the supplementary analyses in electronic supplementary material, S9). At the macroevolutionary scale, the rate of evolution for each of the five morphological traits was estimated on each the 1000 species-level trees for BM and OU models using the *mvMORPH* R package [48]. We assigned to the *A. clarkii* species, for each trait, the mean trait value of the 53 *A. clarkii* individuals. The best fitting model was determined using the Akaike information criterion corrected for sample size (AICc). We additionally analysed the traits using the phylogenetic Monte Carlo method (from the *pmc* R package [49]) to assess if the phylogenies at both scales had sufficient statistical power to distinguish between BM and OU processes. We also ran the two models with *fitContinuous (*from the *geiger* R package [26]) to quantify the amount of parameter uncertainty due to the rather small size of the phylogeny. The *pmc* and *fitContinuous* analyses were both ran on a consensus tree built using Treeannotator [50] from the distribution of 1000 species-level trees.

### (e) Predicting macroevolution from microevolution

We used two different approaches, one for molecular sequences and one for morphological traits, to predict the rates of evolution between clownfishes species from rates of evolution inside *A. clarkii*.

For *RH1* molecular sequences obtained at both microevolutionary and macroevolutionary scales, we expect that a large amount of the polymorphism observed at present will not be maintained through long evolutionary timescales. Considering that each *A. clarkii* individual could be an haplotype that might get fixed in this species by drift, we can artificially simulate macroevolution by pruning all but one *A. clarkii* individual in the combined tree containing all clownfish species and *A. clarkii* individuals. This approach allowed us to predict the species-level rate of evolution of *A. clarkii* by estimating the rate on the terminal branch remaining with a single *A. clarkii* individual (figure 2). We repeated the analysis 53 times, keeping each time only one of the 53 *A. clarkii* individuals, to account for the variability in the choice of individual selected. This molecular evolutionary rate estimated from the branch of the *A. clarkii* was then compared with the rate of evolution estimated from the tree containing all the species of clownfishes.

For morphological traits, we used simulations to determine whether parameters estimated at the microevolutionary scale can predict the rate of evolution of the mean species trait at the macroevolutionary scale. Our simulations were based on three parameters: (1) the trait variance *s^2^*, (2) the generation time *τ*, and (3) the population size *N*_e_. These parameters were either estimated from our empirical data or obtained from the literature (electronic supplementary material, S2). The simulations can be seen as long-term approximation of the approaches of Jones *et al*. [51,52] and Revell [19], which allow us to use normally distributed phenotypic traits at the population level to evolve macroevolutionary rates of evolution (see electronic supplementary material, S2). Our simulations consist of drawing at each generation *N_e_* individuals from a normal distribution (defined by the mean and the variance of the species trait). While the intraspecific variance (*s*^2^) is assumed to be constant, the mean is obtained as the empirical mean of the trait values of the previous generation. The mean of the trait is thus evolving stochastically from one generation to another [22]. By repeating this process across many generations along the species tree, we generated changes in the species trait values at macroevolutionary timescales (see electronic supplementary material, S2 for more details). We then fitted OU and BM models on the simulated species trait using the *mvMORPH* package [48] and we ranked the models by AICc. We performed 1000 simulations for each trait using the distribution of 1000 species-level trees. We first compared if the best fitting model for simulations was the same than the best fitting model for the empirical data. Then, for each trait, we compared the rate of evolution (*σ*^2^) inferred from the 1000 simulations with the range of rate values obtained from the empirical trait (electronic supplementary material table S4, figure 2). To do this, we computed the probability that our microevolution simulations correctly predict the macroevolutionary rate, e.g. how many simulations provided a rate of evolution falling in the 95% confidence interval of empirical rate values. We also ran specificity tests, to show that our microevolutionary simulations do not predict rates of macroevolution when the population size is artificially smaller or larger than the population size estimated from *A. clarkii* data (electronic supplementary material, table S2).

Our approach relies on the fact that clownfishes are a very homogeneous group of fishes with species sharing a large number of morphological and behaviour traits in common, such as their symbiosis with anemone that structures their life history [53] or their very high self-recruitment rates [54]. *A. clarkii* as a representative of clownfishes species was chosen because its populations reflect some diversity in host usage and distribution range and it is not a specialist species with a too narrow range (such as *A. chrysogaster, A. omanensis, A. mccullochi*).

### (f) Comparison between selective pressures at micro- and macroevolutionary scales

We first estimated whether *RH1* was under positive selection at both micro- and macroevolutionary scales with a McDonald–Kreitman test comparing the amount of variation within *A. clarkii* to the divergence to another species in the tree (*A. ocellaris*) using the approach of Egea *et al.* [55] (implemented in the website http://mkt.uab.es/). We then estimated the non-synonymous–synonymous substitution rate ratio (dN/dS) for each of the 251 amino acid sites using a site model [56] implemented in *SlimCodeML* (v. 2014-02-11 [57]). This analysis was replicated for the 1000 trees at the species level and the 1000 trees of *A. clarkii* separately. Finally, we tested with a Wilcoxon signed-rank tests whether the sites detected under positive selection were also associated with water depth in *A. Clarkii* and in all the other species.

## Supplementary Material

Supplementary Methods

## Supplementary Material

Figure S1

## Supplementary Material

Figure S2

## Supplementary Material

Figure S3

## Supplementary Material

Figure S4

## Supplementary Material

Figure S5

## Supplementary Material

Table S1

## Supplementary Material

Table S2

## Supplementary Material

Table S3

## Supplementary Material

Table S4

## Supplementary Material

Table S5

## Supplementary Material

Table S6

## Supplementary Material

Table S7

## Data accessibility

We made all new molecular data available in GenBank (see the electronic supplementary material and table 2). All the other datasets are available in previously published articles.
