# Supplementary material for: Clownfishes evolution below and above the species level
Source: Proc Biol Sci. 2018 Feb 21;285(1873):20171796. doi: 10.1098/rspb.2017.1796 (PMC5832698; doi:10.1098/rspb.2017.1796)
Supplement: Supplementary Methods [file rspb20171796supp1.docx]

**Clownfishes evolution below and above the species level**

**- Electronic Supplementary Material -**

**Sup. Mat. S1. Morphometric analysis and description of the trait studied**

We placed 12 landmarks on all clownfish pictures, including the sampled *A. clarkii* individuals with the package *Geomorph* [1] in R 3.0.2⁠. Using those landmarks (Figure S2), we measured five key morphological traits:

The body ratio has been used in numerous studies as it is directly related to the hydrodynamic of the fish [2-5]. A more streamlined body is often associated with high flow regimes (reviewed in [6]). For example, deep-bodied species of rabbitfishes are found on reef fronts and elongated species on reef flats where the current is stronger [2]. Deep-bodied fishes might also have a higher maneuverability, which help them to escape predators and to explore complex environment [7-9].

Similarly to the body ratio, the head ratio is a major trait characterizing the fish shape. It is related to hydrodynamic and swimming performances, especially maneuverability [3,10-13]. Head depth plays also a variety of roles in the sensory capabilities providing the potential for large and/or high eyes [14].

The snout angle is also related to the hydrodynamic [10] and to feeding behaviour that may vary between different habitats at different depth: as tubular or longer snout allows easier access to pockets of algae growing in the coral reef [15]. The length of the snout affects trophic and sensory capabilities. For instance, it influences the abilities of fishes to detect and catch preys [14,16-19].

The peduncle factor is related to the energy given to the caudal fin and it is one of the most studied characters related to the propulsion abilities and swimming performance of the fish [20]. For instance, deeper caudal peduncle has been shown to be related to high-flow environments in *P. reticulate* [21]. Related traits such as the narrowest point on caudal penduncle divided by body depth has been used as a proxy for swimming abilities [14].

The eye heigth ratio is also often recorded because it is directly associated with visual capacities and trophic level [17,22]. The vertical position of the eye is susceptible to change in the water column because predators might be more frequent either above or below the fish depth.

**Sup. Mat. S2. Simulations**

Our simulations were based on three parameters: (1) the trait variance *s^2^*, (2) the generation time τ, and (3) the population size *N_e_*. (1) The trait variance was measured from the 53 *A. clarkii* individuals. Using a permutation test, we showed that 53 individuals is sufficient to get a robust estimate of the trait variance for *A. clarkii* (see Sup. Mat. S8 and Figure S3 for more details). (2) We obtained estimates of generation time of 5 years in *A. perculla* from the literature [23] (but given the low number of references found for clownfishes and our own personal observations, we considered generation time to range between 3 and 7 years. These relative long generation times compared to other coral reef fishes are essentially due to the waiting time to reach sexual maturity (determined by the social structure of each group of clownfish individuals living in a single anemone) and are also related to the exceptional life span of clownfishes [23]. (3) To obtain effective population size estimate (*N_e_*) we reconstructed a tree of the 41 *A. clarkii* individuals (for which the two mitochondrial markers have been sequenced) using coalescent priors and constant population size in BEAST 2.3 [24] (we used the same procedure as described in Sup. Mat. S5). This subsampling of the dataset allowed us to be more precise in the estimation of the *N_e_* parameter. We calibrated the root of the coalescent tree using a normal prior (mean = 1.51 Myrs, sd = 0.27 Myrs) corresponding to the crown node of *A. clarkii* in the distribution of 1,000 species-level trees. We ran 10 × 10^6^ generations of Markov chain Monte Carlo (MCMC), sampling model parameters every 1,000 generations. This analysis provided the estimate of the "popsize" parameter representing the product of effective population size (*N_e_*) and the generation length (τ). We thus obtained *N_e_* using N_e_ = popsize/τ. To account for the variability in both τ and the popsize parameter, we computed *N_e_* for each simulation from τ and popsize both drawn from their respective uniform distribution (τ = [3,7], popsize = [1.0928, 3.7512]). The range of resulting effective population sizes was log(N_e_) = 11.5–14.5.

We assumed that *N_e_* was constant and similar in all species and that all individuals were replaced by new ones at each generation. Although our simulations are related to previous work [25-27], we simplified the approach because i) we do not have information on the number of loci encoding each trait, ii) we do not know the variance of the effects (sensu [25]) of each mutation for the traits measured for the clownfishes, and iii) our simulations ran for millions of generations, which allow us to look at the long term effects of the process and discard the fine scale mechanisms involved at each generation. At such scale, the fitness landscape may considerably change [28], we thus considered that the fitness function was uniform across the values of the phenotypic trait. In agreement with a large body of literature (e.g. [25,29]), we considered that phenotypes were normally distributed within a population. The trait values across individuals of one species at a given time *t*, here indicated by *x_t_* = [x_1_, …, x_Ne_], were assumed to be normally distributed with sample mean *m_t_* , which represents the mean species trait value, and variance *s^2^*. At each generation, new trait values for all individuals were drawn from a normal distribution with mean equal to the sample mean at the previous generation, whereas the variance was kept constant such that:

 (1).

The sample mean *m_t+1_* is stochastically different from *m_t_*, and the difference is a function of the variance *s^2^* and population size *N_e_* :

 (2).

The difference *m_t+1_* – *m_t_* represents the amount of change in the species trait value that is accumulated in one generation. By repeating this process across many generations, we generated changes in the species trait values at macroevolutionary time scales. Thus, under this model, phenotypic evolution at the species level is mechanistically determined by changes in trait values among the individuals of the species. We simulated the evolution of traits based on equation (2) along the dated phylogeny of all clownfish species. For some of the traits (head ratio, eye height and snout angle), analyses conducted on the species tree (macroevolutionary level) selected OU models against BM (Table S4), suggesting that phenotypes evolve under a constrained process, where the trait values is favored around an optimum. To account for this, we constrained the simulations for those traits by imposing empirical boundaries to the species trait values (sample means). The empirical boundaries were obtained from the variance (*s^2^*) of those traits estimated from the parameters of the OU process inferred at a species-level. Whenever the species values fell outside this range they were reflected back in the simulations.

We observed a good match between the empirical macroevolutionary rates and the macroevolutionary rates predicted by our simulations (see Results). We ran additional analyses to verify that these results did not simply derive from the large confidence intervals around the predicted rate obtained through simulations, i.e. from poor precision. Thus, we ran new simulations assuming the population size to be significantly smaller or significantly larger than that inferred from molecular data, while maintaining the same magnitude of variation in N_e_ as in the original simulations. We sampled the effective population sizes from the ranges log(N_e_) = 8.5–11.5 (smaller population size) and log(N_e_) = 14.5–17.5 (larger population size).

**Sup. Mat. S3. Field sampling**

To reduce the induced stress in sampled individuals, we designed a device to take in-situ standardized underwater pictures of the individuals. The device was composed of two metal frames fixed to one another by a hinge. One frame had a handle and a black background on which a scale was placed. On each frame, we stretched a transparent polyurethane film. The films were flexible enough so that a fish could be held still between both films in a flat position while we took a picture with an underwater camera. We caught the largest fish of the anemone to ensure that we sampled only mature individuals.

**Sup. Mat. S4. Lab procedures**

We amplified a 753 bp fragment of the rhodopsin gene (*RH1*) for all clownfish species and *A. clarkii* individuals with the DamselRh1F1b and DamselRh1R1b primers [30]⁠. The 25 μL polymerase chain reaction (PCR) reaction mixture contained c. 50 ng of DNA template, 2.5 μL GoTaq buffer, 3 μL dNTPs (2.5 μm), 1 Mg Cl_2_ (25 μm), 1 μL of both forward and reverse primer and 0.3 μL of Taq polymerase (GoTaq DNA Polymerase, Promega, Madison, WI, USA). The samples were incubated for 3 min at 94°C, followed by 30 cycles of 1 min at 94°C, 55 sec of annealing at 55°C, and 45 sec at 72°C. The last cycle was followed by a 5 min extension at 72°C. We purified the amplification products with the QIAquick PCR Purification Kit (Qiagen), and sequenced the forward and reverse strands with the Big Dye 3.1 Terminator cycle sequencing kit (Applied Biosystems, Foster City, CA, USA), according to the manufacturer’s instructions, and separated the products on an ABI Prism 3100 genetic analyser (Applied Biosystems). We downloaded the *RH1* sequences of three Pomacentridae species available on GenBank to use as outgroup taxa in the phylogenetic analyses. We amplified the *cytB* fragment (1155 bp) as in [31] and the *CR* (444 bp) as in [32].

For all markers, we checked visually the sequence chromatograms for ambiguous bases. As the following analyzes require homozygous individuals in the *RH1* gene, we discarded the data from samples that showed double peaks in the chromatogram from all analyzes. We removed bases from the sequences that had more than 5% sequencing error probability and built consensus sequences with the forward and reverse strands in Geneious 6 (Biomatters, LTD, Auckland, NZ). We aligned the sequences using MAFFT 7.158b with default settings [33]⁠.

**Sup. Mat. S5. Phylogenetic reconstruction of the relationship between *A. clarkii* individuals**

We reconstructed an ultrametric tree of the *A. clarkii* individuals using a coalescent process for the prior on divergence times and a strict clock BEAST 2.1.3 [24] by concatenating the alignments from the two mitochondrial markers sequenced. This tree prior permit to reconstruct the relationships between individuals of the same species. We partitioned the supermatrix by gene and identified the best model of substitution with the *phymltest* function of the *ape* R [34]⁠. We ran four analyzes independently for 10 × 10^6^ generations each, sampling model parameters every 1,000 generations. We assessed the convergence of the Markov chain Monte Carlo (MCMC) searches by looking at log files in Tracer [35] and verified that the effective sample size (ESS) of each parameter was higher than 200. We removed the first 2,500 trees of each run as the burn-in period, and after merging the four runs, we randomly sampled 1,000 trees.

**Sup. Mat. S6. Phylogenetic reconstruction of clownfishes**

We took advantage of a recent phylogenetic analysis of the clownfishes based on seven nuclear markers and inferred in a Bayesian framework [36]⁠. We extracted 1,000 phylogenetic trees from the posterior distribution obtained in this study to take into account phylogenetic uncertainty in our analyses at the macroevolutionary scale. Several species were sampled multiple times in the original phylogeny [36]. We decided to keep a single individual per species for all species other than *A. clarkii* by randomly pruning duplicates from the topology. Unexpectedly, one species, *Amphiprion tricinctus* renders the *A. clarkii* clade polyphyletic [36]. Because we did not identify the specimen ourselves (we received a fin clip), we could not insure its exact identification. We thus decided to remove it from all further analyzes. We performed all tree manipulations in the R environment using the *ape* [34] and *Geiger* [37] packages.

**Sup. Mat. S7. Rates of *RH1* molecular evolution**

To measure the rate of substitution of the *RH1* gene, we first optimized the branch lengths of the *RH1* tree while keeping the tree topology fixed to that of the dated phylogeny. This step was done with the *optim.pml* function of the R package *phangorn* and we applied a GTR+gamma model of DNA evolution. We then summed the lengths of all branches of the *RH1* tree of *A. clarkii* individuals and divided by the sum of the branches length of the dated tree of *A. clarkii*. We did the same for the tree including all the other clownfish species (including one *A. clarkii* individual) to get the mean rate of clownfish molecular evolution.

**Sup. Mat. S8. Permutation test to estimate if the number of individuals inside *A. clarkii* is sufficient to estimate the intraspecific variance of traits.**

We performed permutation tests to assess if the variance of the traits in *A. clarkii* was biased depending on the number of individuals sampled. For each trait, we estimated the variance from 2 to 53 individuals randomly sampled in the pool of 53 *A. clarkii* individuals. We replicated 100 times this analysis in order to account for potential traits variations between sampled individuals. Our results strongly suggest that the variance of each trait is accurately estimated in *A. Clarkii* (Figure S3).

**Sup. Mat S9. The difference between morphological rates of evolution above and below the species level.** We fitted Brownian Motion (BM) and Ornstein-Uhlenbeck (OU) models of evolution with the five morphological traits on the 1,000 phylogenies of *A. clarkii* individuals using the *mvmorph* package [38]; we found a large difference of rate of evolution between *A. clarkii* and the other clownfish species. For the best fitting model (OU for all traits at the microevolutionary rates, Table S7) - the rates of evolution were always higher in *A. clarkii* individuals than among clownfish species (r_micro-_ = 146.63 [9.46, 7402.46] against r_macro_ = 0.269 [0.20, 0.40] for body ratio, r_micro_ = 396.24 [18.76, 18566.07] against r_macro_ = 0.63 [0.35, 48.92] for head ratio, r_micro_ = 3122.75 [61.13, 45126.49] against r_macro_ = 0.11 [0.09, 0.14] for peduncle factor, r_micro_ = 438.32 [14.81, 15854.35] against r_macro_ = 1.12 [0.69, 3.01] for eye height and r_micro_ = 573.80 [26.66, 27692.38] against r_macro_ = 0.49 [0.27, 1.11] for snout angle, respectively; Table S7, Figure S4). We found that microevolutionary rates were larger than macroevolutionary rate for whatever the model considered (OU or BM models) across a posterior distribution of 1000 trees, indicating that this result is also robust to uncertainties in tree topology and branching times. We however noted that these estimates of morphological evolution are extremely large and we cannot be sure that this variation is not due to plasticity because our sampling.

Supplementary References

1. Adams DC, Otárola-Castillo E. 2013 Geomorph: an R Package for the Collection and Analysis of geometric morphometric shape data. *Methods Ecol. Evol.* **4**, 393-399.

2. Borsa P, Lemer S, Aurelle D. 2007 Patterns of lineage diversification in rabbitfishes. *Mol. Phylogenet. Evol.* **44**, 427-435.

3. Claverie T, Wainwright PC. 2014 A morphospace for reef fishes: elongation is the dominant axis of body shape evolution. *PLoS One*, **9**, e112732.

4. Dumay O, Tari PS, Tomasini JA, Mouillot D. 2004 Functional groups of lagoon fish species in Languedoc Roussillon, southern France. *J. Fish Biol.* **64**, 970-983.

5. Sibbing FA, Nagelkerke LAJ. 2001 Resource partitioning by Lake Tana barbs predicted from fish morphometrics and prey characteristics. *Rev. Fish Biol. Fish.* **10**, 393-437.

6. Langerhans RB. 2008 Predictability of phenotypic differentiation across flow regimes in fishes. *Integr. Comp. Biol.* **48**, 750-768.

7. Webb PW, Weihs D. 1986 Functional locomotor morphology of early life history stages of fishes. *Trans. Am. Fish. Soc.* **115**, 115-127.

8. Goatley CHR, Bellwood DR. 2009 Morphological structure in a reef fish assemblage. *Coral Reefs* 28, 449-457.

9. Holmes T, McCormick M. 2010 Size-selectivity of predatory reef fish on juvenile prey. *Mar. Ecol. Prog. Ser.* **399**, 273-283.

10. Webb PW. 1984 Body form, locomotion, and foraging in aquatic vertebrates. *Am. Zool.* **24**, 107-120.

11. Nanami, A. 2007 Juvenile swimming performance of three fish species on an exposed sandy beach in Japan. *J. Exp. Mar. Biol. Ecol*. **348**, 1-10.

12. Collar DC, Wainwright PC, Alfaro ME. 2008 Integrated diversification of locomotion and feeding in labrid fishes. *Biol. Lett.* **4**, 84-86.

13. Li D, Hu W, Wang Y, Zhu Z, Fu C. 2009 Reduced swimming abilities in fast-growing transgenic common carp *Cyprinus carpio* associated with their morphological variations. *J. Fish Biol.* **74**, 186-197.

14. Bellwood DR, Goatley CHR, Brandl SJ, Bellwood O. 2014 Fifty million years of herbivory on coral reefs: fossils, fish and functional innovations. *Proc. R. Soc. B* **281**, 20133046.

15. Woodland DJ. 1990 Revision of the fish family Siganidae with descriptions of two new species and comments on distribution and biology. *Indo-Pacific Fishes* **19**.

16. Brandl SJ, Bellwood DR. 2013 Pair formation in the herbivorous rabbitfish *Siganus doliatus*. *J.* *Fish Biol*. **82**, 2031.2044.

17. Brandl SJ, Bellwood DR. 2013 Morphology, sociality, and ecology: can morphology predict pairing behavior in coral reef fishes? *Coral Reefs* **32**, 835-846.

18. Schmitz L, Wainwright PC. 2011 Ecomorphology of the eyes and skull in zooplanktivorous labrid fishes. *Coral Reefs* **30**, 415-428.

19. Brandl SJ, Robbins WD, Bellwood DR. 2015 Exploring the nature of ecological specialization in a coral reef fish community: morphology, diet and foraging microhabitat use. *Proc. R. Soc. B***282**, 20151147.

20. Fisher R, Hogan JD. 2007 Morphological predictors of swimming speed: a case study of pre-settlement juvenile coral reef fishes. *J. Exp. Biol*., **210**, 2436-2443.

21. Hendry AP, Kelly ML, Kinnison MT, Reznick DN. 2006 Parallel evolution of the sexes? Effects of predation and habitat features on the size and shape of wild guppies. *J. Evol. Biol.* **19**, 741-754.

22. López-Fernández H, Arbour JH, Winemiller K, Honeycutt RL. 2013 Testing for ancient adaptive radiations in Neotropical cichlid fishes. *Evolution* **67**, 1321-1337.

23. Buston PM, García MB. 2007 An extraordinary life span estimate for the clown anemonefish *Amphiprion percula*. *J. Fish Biol.***70**, 1710-1719.

24. Bouckaert R, *et al.* 2014 BEAST 2: a software platform for Bayesian evolutionary analysis. *PLoS Comput. Biol.* **10**, e1003537.

25. Revell, LJ. 2007 The G matrix under fluctuating correlational mutation and selection. Evolution, **61**, 1857-1872.

26. Jones AG, Arnold SJ, Bürger R. 2003 Stability of the G-matrix in a population experiencing pleiotropic mutation, stabilizing selection, and genetic drift. *Evolution* **57**, 1747-1760.

27. Jones AG, Arnold SJ, Bürger R. 2004 Evolution and stability of the G-matrix on a landscape with a moving optimum. *Evolution* **58**, 1639-1654.

28. Svensson E, Calsbeek R. (Eds.) 2012 The adaptive landscape in evolutionary biology. Oxford University Press.

29. Lande R. 1979 Quantitative genetic analysis of multivariate evolution, applied to brain: body size allometry. *Evolution* **33**, 402-416.

30. Hofmann CM. *et al.* 2012 Opsin evolution in damselfish: convergence, reversal, and parallel evolution across tuning sites. *J Mol. Evol.* **75**, 79-91.

31. Quenouille B, Bermingham E, Planes S. 2004 Molecular systematics of the damselfishes (Teleostei: Pomacentridae): Bayesian phylogenetic analyses of mitochondrial and nuclear DNA sequences. *Mol. Phylogenet. Evol.* **3**, 66-88.

32. Timm J, Figiel M, Kochzius M. 2008 Contrasting patterns in species boundaries and evolution of anemonefishes (Amphiprioninae, Pomacentridae) in the centre of marine biodiversity. *Mol Phylogenet Evol* **49**, 268-276.

33. Katoh K, Standley DM. 2013 MAFFT multiple sequence alignment software version 7: improvements in performance and usability. *Mol. Biol. Evol.* **30**, 772-780 (2013).

34. Paradis E, Claude J, Strimmer K. 2004 APE: Analyses of Phylogenetics and Evolution in R language. *Bioinformatics* **20**, 289-290.

35. Drummond AJ, Rambaut A. 2007 BEAST: Bayesian evolutionary analysis by sampling trees. *BMC Evol. Biol.* **7**, 214 (2007)

36. Litsios G, Pearman PB, Lanterbecq D, Tolou N, Salamin N. 2014 The radiation of the clownfishes has two geographical replicates. *J. Biogeogr.* **41**, 2140-2149.

37. Harmon LJ, Weir JT, Brock CD, Glor RE, Challenger W. 2008 GEIGER: investigating evolutionary radiations. *Bioinformatics* **24**, 129-131.

38. Clavel J, Escarguel G, Merceron G. 2015 mvMORPH: an R package for fitting multivariate evolutionary models to morphometric data. *Methods Ecol. Evol.* **6**, 1311-1319.

39. Lieske E, Myers R. 1994. *Collins Pocket Guide. Coral reef fishes. Indo-Pacific & Caribbean including the Red Sea.* Haper Collins Publishers, 400 p.

40. Allen GR. 1991. *Damselfishes of the world.* Mergus Publishers, Melle, Germany. 271 p.

41. Allen GR. Drew J, Kaufman L. 2008 Amphiprion barberi, a new species of anemonefish (Pomacentridae) from Fiji, Tonga, and Samoa. *Aqua Int. J. Ichthyol.* **14**:105-114.

42. Bacchet P, Zysman T, Lefèvre Y. 2006 *Guide des poissons de Tahiti et ses îles. Tahiti (Polynésie Francaise).* Editions Au Vent des Îles. 608 p.

43. Myers RF. 1991 *Micronesian reef fishes.* Second Ed. Coral Graphics, Barrigada, Guam. 298 p.

44. Allen GR, Drew J, Fenner D. 2010 Amphiprion pacificus, a new species of anemonefish (Pomacentridae) from Fiji, Tonga, Samoa, and Wallis Island. aqua, *Intl. J. Ichthyol.* **16** :129-138.

45. Fricke R, Kulbicki M, Wantiez L. 2011 Checklist of the fishes of New Caledonia, and their distribution in the Southwest Pacific Ocean (Pisces). *Stuttgarter Beiträge zur Naturkunde A, Neue Serie* **4**, 341-463.
