# Supplementary material for: Clownfishes evolution below and above the species level
Source: Proc Biol Sci. 2018 Feb 21;285(1873):20171796. doi: 10.1098/rspb.2017.1796 (PMC5832698; doi:10.1098/rspb.2017.1796)
Supplement: Figure S1 [file rspb20171796supp2.docx]

**Figure S1. Distributions of likelihood ratios (δ) between BM and OU models based on 1,000 simulations using *pmc***. Brown histograms show the likelihood ratios between OU and BM for 1000 traits simulated under BM. Blue histograms show the likelihood ratios between OU and BM for 1000 traits simulated under OU. The red lines indicate the likelihood ratio between OU and BM fitted on the empirical traits. For body ratio and peduncle factor, the large overlap of likelihood ratios show that there is no sufficient statistical power to distinguish between models. For these traits we therefore chose the simplest model (BM). For the other traits, the distributions of likelihood ratios are clearly distinct and the red line falls within the range of the OU model, which suggest that we have sufficient statistical power to distinguish between the two models.
