# Supplementary material for: Clownfishes evolution below and above the species level
Source: Proc Biol Sci. 2018 Feb 21;285(1873):20171796. doi: 10.1098/rspb.2017.1796 (PMC5832698; doi:10.1098/rspb.2017.1796)
Supplement: Figure S2 [file rspb20171796supp3.docx]

**Figure S2. Description of the five morphological traits used in the study.** Each trait is described according to the landmarks on the picture. Body ratio: The body ratio was calculated by dividing the standard length (distance between the snout *(1)* and the base of the tail, which is exactly between (*8)* and (*9)*) by the body depth (segment between the base of the pelvic fin (*11)* and the base of the spinous dorsal fin *(6)*). Head ratio: The head ratio represents the standard length divided by the head length (distance between the snout *(1)* and the origin of the opercular *(12)*). Peduncle factor: The peduncle depth factor was calculated by dividing the body depth by the caudal peduncle depth (distance between the posterior end of the dorsal fin base *(7)* and the posterior end of the anal fin base *(10)*). Eye height ratio: The eye height ratio is the ratio between the body depth and the eye height, which is the distance between the center of the eye (center of gravity of *(2, 3, 4, 5))* and the point where the line parallel to the body depth (passing through the center of the eye) meets the line parallel to the standard length (passing through *(11))*. Snout angle: The snout angle is the angle calculated between the standard length and the line that passes through the snout tip *(1)* and the base of the spinous dorsal fin *(6)*).
