# Supplementary material for: Clownfishes evolution below and above the species level
Source: Proc Biol Sci. 2018 Feb 21;285(1873):20171796. doi: 10.1098/rspb.2017.1796 (PMC5832698; doi:10.1098/rspb.2017.1796)
Supplement: Figure S3 [file rspb20171796supp4.docx]

**Figure S3. Permutation tests suggesting that the number of individuals is sufficient to accurately estimate the variance of traits in *A. clarkii.*** Each boxplot represent 100 estimations of variance from 2 to 53 individuals randomly chosen in the microevolutionary dataset of 53 *A. clarkii* individuals.
