# Supplementary material for: Clownfishes evolution below and above the species level
Source: Proc Biol Sci. 2018 Feb 21;285(1873):20171796. doi: 10.1098/rspb.2017.1796 (PMC5832698; doi:10.1098/rspb.2017.1796)
Supplement: Figure S4 [file rspb20171796supp5.docx]

**Figure S4. Comparison between the microevolutionary rates, the macroevolution rates estimated from empirical data and the macroevolution rates simulated (based on intraspecific parameters) for the five morphological traits.** The columns The columns (micro) in yellow and (macro) in black correspond to the estimates of the micro- and the macroevolutionary rates observed in the data (respectively), and (macro_P) in blue, represent the predicted macroevolutionary rate of the simulations based on effective population size, trait variance and generation time (described in details in Sup. Mat. S2). These violon plots represent the variation across the 1,000 trees. For each trait, simulated and empirical rates of evolution (σ^2^) were obtained from best fitting model chosen only from the empirical data (BM or OU, Table S4, Table S7). The predicted and observed distributions of macroevolutionary rates did not differ significantly in any of the traits (P > 0.05).
