# Supplementary material for: Clownfishes evolution below and above the species level
Source: Proc Biol Sci. 2018 Feb 21;285(1873):20171796. doi: 10.1098/rspb.2017.1796 (PMC5832698; doi:10.1098/rspb.2017.1796)
Supplement: Figure S5 [file rspb20171796supp6.docx]

**Figure S5. Median joining network analysis from POPART (Population Analysis with Reticulate Trees, Bandelt *et al.* 1999; http://popart.otago.ac.nz) based on two mitochondrial markers, showing that *A. clarkii* individuals have different haplotypes and have no particular geographical clustering.** This analysis evaluates the divergence between individuals in our dataset and their sampling sites location. This analysis suggested that the individuals of *A. clarkii* sampled represent highly divergent haplotypes and likely come from different populations. As we sampled one individual per anemone in different reefs in the center of the distribution of *A. clarkii*, we expect to have sampled a large proportion of the genetic and morphological variance of the species.
