# Supplementary material for: Clownfishes evolution below and above the species level
Source: Proc Biol Sci. 2018 Feb 21;285(1873):20171796. doi: 10.1098/rspb.2017.1796 (PMC5832698; doi:10.1098/rspb.2017.1796)
Supplement: Table S1 [file rspb20171796supp7.docx]

**Table S1. References of the samples used for estimating morphological rates above the species** level. (CAS: California Academy of Sciences, AMNH: American Museum of Natural History, New York, NMNH: National Museum of Natural History, Smithsonian Institution, GO: fieldwork pictures obtained directly from John E Randall's  http://pbs.bishopmuseum.org/, MCZ: Museum of Comparative Zoology, Harvard University, Cambridge, Massachusetts).
