# Supplementary material for: Clownfishes evolution below and above the species level
Source: Proc Biol Sci. 2018 Feb 21;285(1873):20171796. doi: 10.1098/rspb.2017.1796 (PMC5832698; doi:10.1098/rspb.2017.1796)
Supplement: Table S2 [file rspb20171796supp8.docx]

**Table S2. Specificity tests showing that there is no link between micro and macroevolution if the effective population size is artificially smaller or larger than the size estimated from *A. clarkii* data.** In the table, “Yes” means that we cannot reject the hypothesis that microevolutionary simulations predict empirical macroevolutionary rates (P>0.05) and “No” means that microevolutionary simulations do not predict a link between micro and macroevolution (P<0.05). Rates of evolution (r) and P-values are the result of 1,000 simulations. Using the empirically estimated population size macroevolutioanry rates are predicted in all simulations. Simulations run with artificially smaller or larger populations size did not predict macroevolutionary rates.
