# Supplementary material for: Clownfishes evolution below and above the species level
Source: Proc Biol Sci. 2018 Feb 21;285(1873):20171796. doi: 10.1098/rspb.2017.1796 (PMC5832698; doi:10.1098/rspb.2017.1796)
Supplement: Table S3 [file rspb20171796supp9.docx]

**Table S3. List of the Genbank accession numbers of the newly sequenced *RH1* in this study for the 26 species of clownfishes and the mean water depth used from fishbase (except *A. clarkii*, for which we used field data).**
