# Supplementary material for: Clownfishes evolution below and above the species level
Source: Proc Biol Sci. 2018 Feb 21;285(1873):20171796. doi: 10.1098/rspb.2017.1796 (PMC5832698; doi:10.1098/rspb.2017.1796)
Supplement: Table S4 [file rspb20171796supp10.docx]

**Table S4.** **Rates of morphological evolution at interspecific level can be estimated from simulations based on intraspecific parameters.** The table displays the results of the analysis of trait macroevolution based on empirical data (left) and on the traits simulated under intraspecific parameters (right). AICc and model parameters were summarized across 1,000 trees (median and 95% confidence intervals provided in brackets). For both empirical and simulated traits, we fitted BM and OU models using maximum likelihood and compared their fit using AICc. We provide the percentage of the 1,000 trees supporting each model according to the AICc (the best fitting model is bolded). The estimated model parameters are: the rate of evolution (σ^2^, noted r_macro_ in the main text) and the strength of the OU process (α). The same best fitting model was retrieved from simulations for all traits except for eye height, i.e. in this trait BM and OU models were equally likely. We computed the probability (P) that simulations correctly predict the macroevolutionary rate, e.g. how many simulations provided a rate of evolution falling in the 95% confidence interval of empirical rate values (P > 0.05 indicates that the predicted macroevolutionary rate is not significantly different than the macroevolutionary rate inferred from the clownfish empirical data). For more consistency, traits values were standardized: we computed the difference between each value and the mean value of the trait. This difference was then divided by the standard deviation of the trait values.
