# Supplementary material for: Clownfishes evolution below and above the species level
Source: Proc Biol Sci. 2018 Feb 21;285(1873):20171796. doi: 10.1098/rspb.2017.1796 (PMC5832698; doi:10.1098/rspb.2017.1796)
Supplement: Table S5 [file rspb20171796supp11.docx]

**Table S5. 95% Confidence intervals around BM and OU parameters estimated on the consensus tree using *fitContinuous* (*geiger* R package).** Rates of morphological evolution and confidence intervals around the rates are similar to those estimated with different topologies, suggesting that the small size of our phylogeny is not affecting our results.
