# Supplementary material for: Clownfishes evolution below and above the species level
Source: Proc Biol Sci. 2018 Feb 21;285(1873):20171796. doi: 10.1098/rspb.2017.1796 (PMC5832698; doi:10.1098/rspb.2017.1796)
Supplement: Table S6 [file rspb20171796supp12.docx]

**Table S6. McDonald Kreitman test.** Number of neutral and non-neutral polymorphisms found at the intraspecific level compared to the number of substitution (divergence) at the interspecific level for *RH1*. This test was significant (χ2 = 5.021; P<0.05), which suggest that *RH1* is under positive selection.
