# Supplementary material for: Clownfishes evolution below and above the species level
Source: Proc Biol Sci. 2018 Feb 21;285(1873):20171796. doi: 10.1098/rspb.2017.1796 (PMC5832698; doi:10.1098/rspb.2017.1796)
Supplement: Table S7 [file rspb20171796supp13.docx]

**Table S7. Rates of morphological evolution below the species level and confidence intervals obtained from different topologies.** These rates are substantially larger than those obtained from the analysis at the species level.
